# Antipruritic Effect of Nalbuphine, a Kappa Opioid Receptor Agonist, in Mice: A Pan Antipruritic

**DOI:** 10.3390/molecules26185517

**Published:** 2021-09-11

**Authors:** Saadet Inan, Nae J. Dun, Alan Cowan

**Affiliations:** 1 Department of Neural Sciences, Center for Substance Abuse Research, Lewis Katz School of Medicine at Temple University, Philadelphia, PA 19104, USA; 2 Department of Pharmacology, Lewis Katz of Medicine at Temple University, Philadelphia, PA 19104, USA; ndun@temple.edu

**Keywords:** nalbuphine, kappa opioid receptor agonist, pruritis, scratching, mice, TAT-HIV, cholestasis, chloroquine, deoxycholic acid

## Abstract

Antipruritic effects of kappa opioid receptor (KOR) agonists have been shown in rodent models of acute and chronic scratching (itchlike behavior). Three KOR agonists, nalfurafine, difelikefalin, and nalbuphine, are in clinical studies for antipruritic effects in chronic itch of systemic and skin diseases. Nalfurafine (in Japan) and difelikefalin (in the USA) were approved to be used in the treatment of chronic itch in hemodialysis patients. The FDA-approved nalbuphine has been used in clinic for over 40 years, and it is the only narcotic agonist that is not scheduled. We aimed to study (a) antiscratch activity of nalbuphine against TAT-HIV-1 protein (controls HIV transcription)-, deoxycholic acid (DCA, bile acid)-, and chloroquine (CQ)-induced scratching in a mouse model of acute itch; and (b) whether the effect of nalbuphine is produced via KORs. First, dose–responses were developed for pruritogens. Mice were pretreated with nalbuphine (0.3–10 mg/kg) and then a submaximal dose of pruritogens were administered and the number of scratching bouts was counted. To study if the antiscratch effect of nalbuphine is produced via KOR, we used KOR knock out mice and pharmacologic inhibition of KORs using nor-binaltorphimine, a KOR antagonist. For this aim, we used CQ as a pruritogen. We found that: (a) TAT-HIV-1 protein elicits scratching in a dose-dependent manner; (b) nalbuphine inhibits scratching induced by TAT-HIV-1, DCA, and CQ dose-dependently; and (c) nalbuphine inhibits scratching induced by CQ through KORs. In conclusion, nalbuphine inhibits scratching elicited by multiple pruritogens.

## 1. Introduction

**Figure 1:**
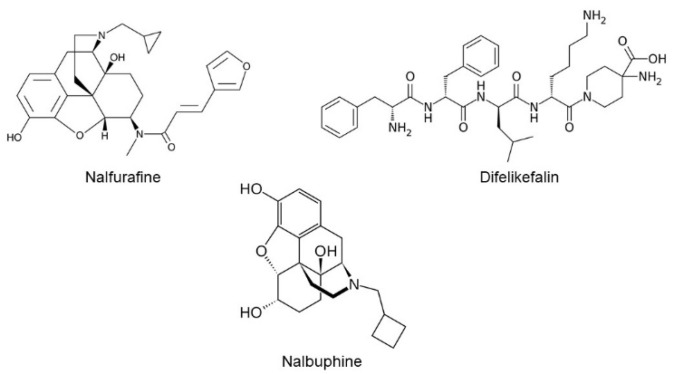
Chemical structures of nalfurafine, difelikefalin, and nalbuphine.

As of today, three kappa opioid receptor (KOR) agonists, nalfurafine (TRK-820, Remich^®^), difelikefalin (CR845, Korsuva™), and nalbuphine (Haduvio™, KOR agonist and a weak mu opioid receptor partial agonist) (Figure 1) are in clinical studies for treating chronic itch of chronic kidney disease, cholestatic liver disease, and atopic dermatitis [1,2,3,4]. Further, nalfurafine was approved in Japan and recently, difelikefalin was approved by the FDA in the USA (https://korsuva.com) (accessed on 2 September 2021) for the treatment of chronic itch in hemodialysis patients. Evidence for the involvement of KORs and for the antipruritic activity of KOR agonists go back to early 1980s. Gmerek and Cowan [5] introduced a rat model that allowed quantitative measurements of scratching (itch like behavior) by intracerebroventricular administration of bombesin, a tetradecapeptide originally isolated from frog skin and a homolog of mammalian gastrin-releasing factor (GRP). Bombesin induced excessive grooming and scratching of the face, head, and neck with the hindpaws in a dose-dependent manner [5]. Later, GRP was identified as one of the mediators for itch transmission at the spinal cord level [6,7]. For the first time, Gmerek and Cowan reported that systemic administration of early benzomorphan KOR agonists (e.g, bremazocine, cyclazocine, ketocyclazocine and pentazocine) significantly reduced bombesin-induced grooming and scratching in rats in a dose-dependent manner in 1984 [8]. Another early piece of evidence was the observation of excessive scratching in monkeys during withdrawal from chronic administration of the KOR agonist U50,488 [9]. Recently, it was shown that B5-I inhibitory neurons express Dynorphin, an endogenous KOR agonist, and inhibit itch sensation at the spinal level [10,11].

Our interest was predominantly in nalbuphine, an FDA-approved analgesic that has been known over 40 years. It is the only narcotic agonist currently unscheduled. Based on GTPgS binding assays, nalbuphine was described as a full agonist on kappa opioid receptors and a weak partial agonist on mu opioid receptors. Nalbuphine both stimulated [35S] GTPgS binding mediated by the mu opioid receptor and inhibited DAMGO-stimulated [35S] GTPgS binding in cultured cells [12,13,14]. The results of the earliest preclinical and clinical studies suggest that nalbuphine is a highly effective analgesic with minimal respiratory depression, inhibition of gastrointestinal transit, analgesic tolerance, physical dependence, and psychotomimetic potential (dysphoria only at doses beyond the therapeutic range) [15]. Hawi et al. [16] showed antiscratch activity of nalbuphine in mice against Substance-P-induced acute scratching. We reported that acute systemic administration of nalbuphine also significantly reduced scratching in mice with chronic contact dermatitis [17]. Chronic itch is a very unpleasant symptom of skin and systemic diseases such as chronic kidney disease, cholestatic liver disease, some cancers, hematologic diseases, psychiatric diseases, and neuropathies, or is idiopathic with no reason. One third of the dermatology patients have chronic itch [18] and, overall, nearly 15% of the general population have this symptom [19,20]. The quality of life of patients with chronic itch is affected seriously. Patients can suffer from sleep deprivation, agitation, depression, suicidal thoughts, and difficulty concentrating [21,22]. Chronic itch is also reported in HIV-infected patients with or without skin conditions [23,24]. Chronic itch could be related common forms of dermatosis, such as seborrheic dermatitis, psoriasis, prurigo nodularis, as well as HIV-associated pruritic popular eruptions, and eosinophilic folliculitis. TAT-HIV-1, a viral protein that controls HIV-transcription, plays an important role in the development of HIV-1 infection as well as in the pathogenesis of complications such as dementia, cardiovascular diseases, and retinal diseases. For example, it was shown that exposure of TAT-HIV-1 induces cytotoxic effects in human brain microvascular endothelial cells [25] and causes apoptotic cell death in retinal pigment epithelial cells [26]. We aimed to study whether nalbuphine would be effective against scratching in mouse models of HIV and cholestasis, as well as chloroquine (CQ)-induced scratching. We first studied whether behind the neck injection of TAT-HIV-1 protein would elicit scratching in mice. We used previously shown induction of scratching by behind the neck injection of a bile acid, deoxycholic acid to study cholestasis [27,28]. We had shown previously that nalfurafine inhibits scratching induced by CQ [29], so lastly, we studied whether nalbuphine, like nalfurafine, would inhibit scratching in mice. We also studied whether antiscratch activity of nalbuphine is produced through KORs in mice injected with CQ. The results of these studies will be beneficial for treatment of HIV- and cholestasis-related chronic itch, as well as itch due to CQ for treatment for malaria in susceptible individuals [30].

## 2. Results

### 2.1. TAT-HIV-1 Induces Scratching and Nalbuphine Inhibits Scratching in a Dose-Dependent Manner

**Figure 2:**
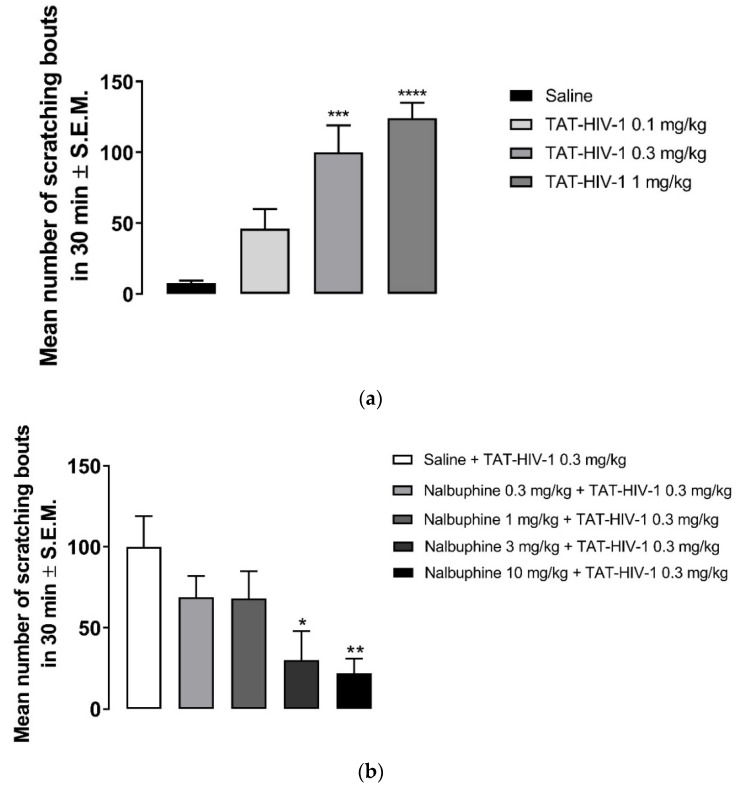

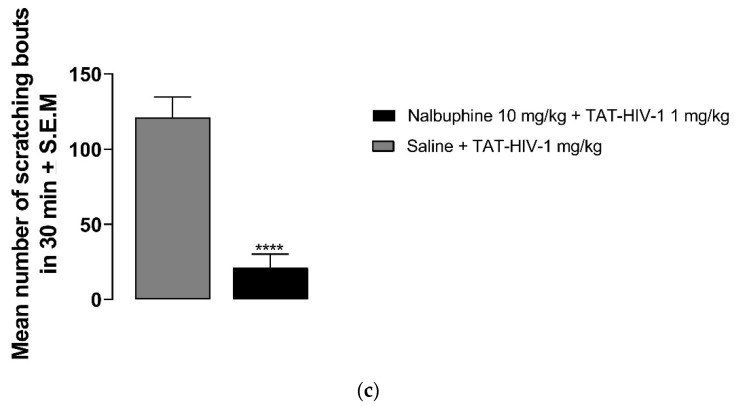
Subcutaneous behind the neck injection of TAT-HIV-1 induces scratching in a dose-dependent manner. (**a**) Mice were administered saline (s.c., flank area) and then 30 min later they were injected with either saline or TAT-HIV-1 (0.1–1 mg/kg, behind the neck). One min following injections, the number of scratching bouts was counted for 30 min. Both TAT-HIV-1 at 0.3 and 1 mg/kg elicited scratching significantly compared to saline (one-way ANOVA followed by Dunnett’s multiple comparison; *** *p* < 0.001, **** *p* < 0.0001; n = 6). Nalbuphine inhibits TAT-HIV-1-induced scratching in a dose-dependent manner; (**b**) Nalbuphine at 3 and 10 mg/kg doses inhibited scratching elicited by submaximal dose of TAT-HIV-1 (0.3 mg/kg) significantly compared to control (one-way ANOVA followed by Dunnett’s multiple comparison; * *p* < 0.05, ** *p* < 0.01; n = 6). Nalbuphine at 10 mg/kg also alleviated scratching induced by maximum dose of TAT-HIV-1 (1 mg/kg); (**c**) (unpaired Student’s *t*-test; **** *p* < 0.0001; n = 6). Swiss–Webster mice were used for these studies.

As seen in Figure 2a, behind the neck (s.c.) administration of TAT-HIV-1 elicited scratching dose-dependently in mice. TAT-HIV-1 at 0.3 and 1 mg/kg doses induced scratching behavior significantly compared to saline (one-way ANOVA followed by Dunnett’s multiple comparison, *** *p* < 0.001, **** *p* < 0.0001). At 1 mg/kg, 124 ± 6 scratching bouts was observed in 30 min. Next, we pretreated (−30 min) mice with either saline or nalbuphine (0.3–10 mg/kg, s.c.) and then a submaximum dose (0.3 mg/kg) of TAT-HIV-1 was injected the nape of the mice. Nalbuphine alleviated TAT-HIV-1-induced scratching in a dose-dependent manner (Figure 2b). Nalbuphine at 3 and 10 mg/kg inhibited scratching significantly compared to control (* *p* < 0.05, ** *p* < 0.01). As seen in Figure 2c, nalbuphine at 10 mg/kg also significantly inhibited scratching elicited by the maximum dose of TAT-HIV-1 (1 mg/kg) (**** *p* < 0.0001).

### 2.2. Nalbuphine Inhibits Deoxycholic Acid-Induced Scratching

**Figure 3:**
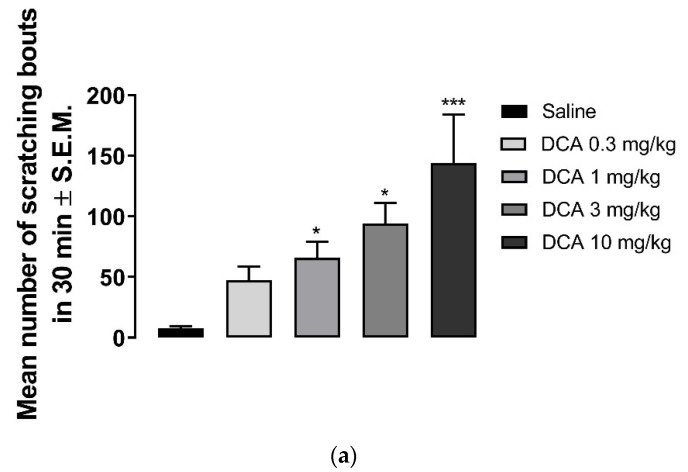

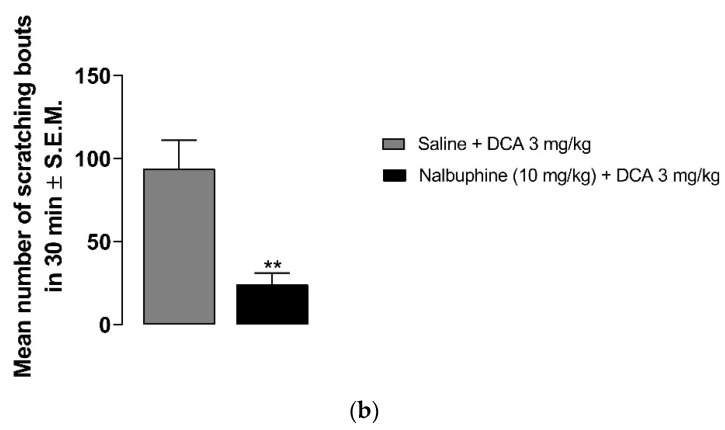
Nalbuphine inhibits DCA-induced scratching. DCA at 1, 3, and 10 mg/kg induces significant scratching compared to saline. (**a**) (one-way ANOVA followed by Dunnett’s multiple comparison; * *p* < 0.05, *** *p* < 0.001; n = 7–8). Pretreatment with nalbuphine 10 mg/kg significantly reduces the number of scratching bouts in 30 min; (**b**) (unpaired Student’s *t*-test; ** *p* < 0.01); n = 6). Swiss–Webster mice were used for these studies.

Deoxycholic acid in doses of 0.3–10 mg/kg induced scratching (Figure 3a). While the number of scratching bouts was 47.5 ± 11 at 0.3 mg/kg, 144 ± 40 scratching bouts was observed at 10 mg/kg dose of DCA. Nalbuphine at 10 mg/kg was administered 30 min before DCA 3 mg/kg to test whether nalbuphine also inhibits DCA-induced scratching. As seen in Figure 3b, nalbuphine significantly reduced scratching bouts induced by DCA (Unpaired Student’s *t*-test; ** *p* < 0.01).

### 2.3. Nalbuphine Inhibits CQ-Induced Scratching through KOR

**Figure 4:**
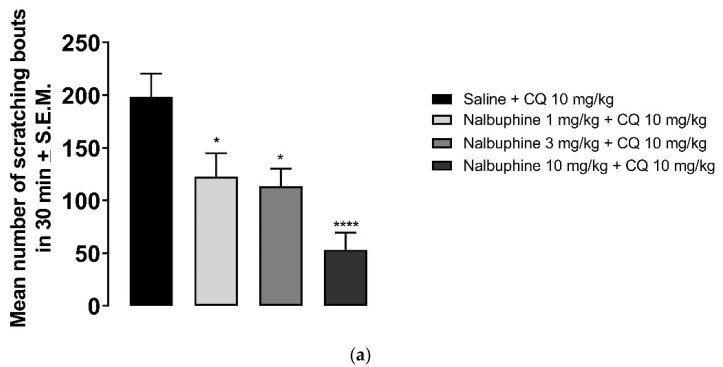

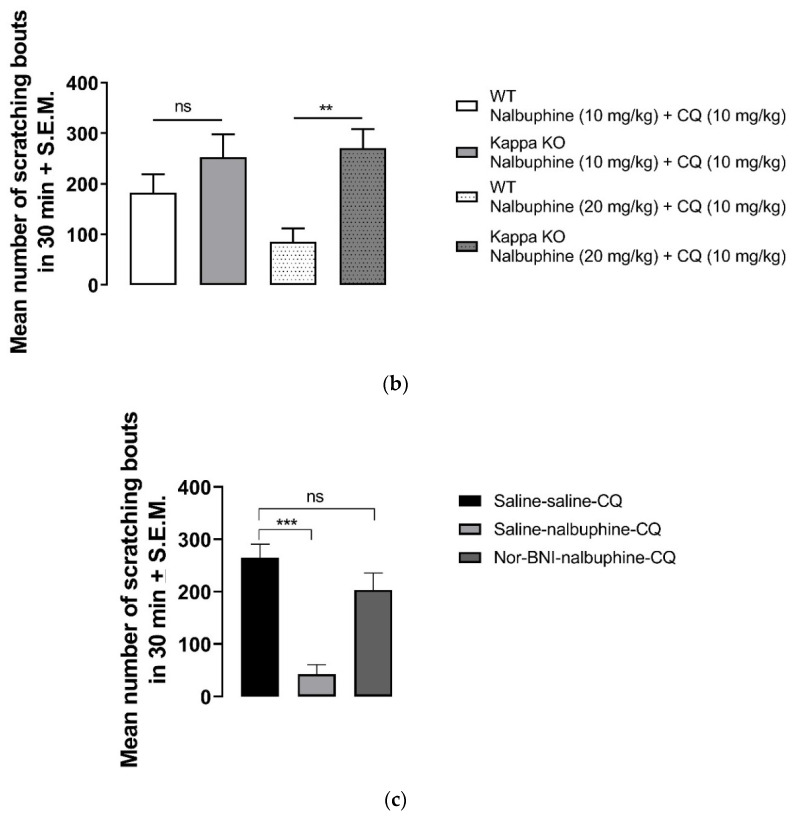
Nalbuphine significantly reduced CQ-induced scratching bouts in a dose-dependent manner. (**a**) Swiss–Webster mice were injected with either saline or nalbuphine (1, 3, or 10 mg/kg). Thirty min later, they were administered a submaximal dose (10 mg/kg, s.c., behind the neck) of CQ. Then, the number of scratching bouts was counted for 30 min. Nalbuphine did not decrease scratching in KOR KO mice; (**b**) Nalbuphine at 10 mg/kg did not have significant effect in C57BL/6J WT mice as in Swiss–Webster. Nalbuphine at 20 mg/kg significantly reduced scratching induced by CQ in WT mice, but not in KOR KO mice. Nalbuphine had no antiscratch effect in Swiss–Webster mice pretreated with nor-BNI; (**c**) Mice were administered with nor-BNI or saline 20 h before the nalbuphine injection. Then, mice received either saline or nalbuphine. Thirty min later, they were injected with CQ. Nalbuphine significantly reduced scratching in mice pretreated with saline the day before; however, no significant effect was observed in mice pretreated with nor-BNI. (one-way ANOVA followed by Dunnett’s multiple comparison; * *p* < 0.05, ** *p* < 0.01, *** *p* < 0.001, **** *p* < 0.0001; n = 7–8).

Nalbuphine (1–10 mg/kg) significantly inhibited scratching bouts induced by submaximal dose of CQ, as seen in Figure 4a (* *p* < 0.05, *** *p* < 0.001). Next, we studied whether the antipruritic effects of nalbuphine function via through KORs. WT C57BL/6J mice did not respond to nalbuphine 10 mg/kg in the same manner as did Swiss–Webster mice. As seen in Figure 4b, nalbuphine at 10 mg/kg did not significantly reduced the number of scratching bouts induced by CQ. Then, we tried nalbuphine at 20 mg/kg in C57BL/6J mice. The animals moved freely and did not show behavioral depression against a higher dose of nalbuphine. A significant decrease in scratching was observed with nalbuphine on WT mice. However, nalbuphine did not have any effect on KOR KO mice. The pharmacological inhibition of KORs using nor-BNI also showed similar results. In Swiss–Webster mice pretreated with nor-BNI, nalbuphine did not inhibit CQ-induced scratching (Figure 4c).

## 3. Discussion

The results of these studies clearly indicate that nalbuphine, a kappa opioid receptor agonist, alleviate scratching bouts elicited by chemically different pruritogens, TAT-HIV-1 protein, DCA, and chloroquine. Additionally, we have shown that antiscratch effect of nalbuphine in CQ-induced scratching operates via KORs. Here, for the first time we report that TAT-HIV-1 protein induces scratching behavior in mice in a dose-dependent manner when it is given s.c. behind the neck. As expected, DCA also elicited scratching in our study in mice, as previously reported [27,28]. Nalbuphine inhibited scratching induced by CQ as well.

As seen in Figure 2a, the TAT-HIV-1 protein elicited scratching behavior dose-dependently. The highest dose (1 mg/kg) that we used caused average of 124 ± 6 scratching bouts in 30 min. Pretreating mice with nalbuphine (10 mg/kg) significantly reduced scratching to an average of 20 ± 7 (Figure 2b). Previously, it was reported that U50,488, a KOR agonist, inhibited HIV-1 expression in human microglia and macrophages [31,32]. Additionally, neurotoxicity-induced by HIV-1 was suppressed by U50, 488 in human microglia cell culture [32]. In the same study, the authors additionally reported that neuroprotective effect of U50,488 was through KORs. In another study, inhibition of TAT-HIV-1-induced production of chemokine, chemoattractant protein-1 in human astrocytes by U50,488 was reported via KORs since nor-BNI blocked the effect of U50,488 [33]. Skin diseases can develop in almost 90% of HIV-positive patients [24,34,35]. Prevalence of chronic pruritus in HIV-positive patients has been reported as 31% from a study conducted in Spain [36] and 45% from a study conducted in southeastern United States [37]. In the later study, itch was found to have a significant negative impact on quality of patient life. In a study including over 4000 HIV patients, it was shown that African Americans are at a higher risk of developing pruritic skin conditions compared to race-matched controls and white patients [38]. TAT-HIV-1 protein might be a contributing factor in the pathogenesis of chronic itch in HIV-positive patients. HIV-infected patients can be targeted with the antipruritic effect of nalbuphine in clinical studies.

As expected, the behind the neck injection of DCA induced scratching beginning at 1 mg/kg in a dose-dependent manner (Figure 3a). Nalbuphine 10 mg/kg was studied against one dose of DCA (3 mg/kg) and it was found that nalbuphine significantly reduced DCA-induced scratching bouts, as shown in Figure 3b. Pathogenesis of chronic pruritus of cholestasis is still elusive. It was reported that multiple mediators contribute in the same way as bile acids (by activating TGR5 receptors), endogenous opioid peptides, activation of autotoxin [39,40]. Still, there is no effective treatment against pruritus of cholestasis; however, promising clinical studies with KOR agonists are being developed (https://www.caratherapeutics.com/our-pipeline/; https://www.trevitherapeutics.com/pipeline/ (accessed on 10 August 2021)). Golpanian et al. [41] reported that butorphanol, a partial KOR agonist and mu opioid receptor antagonist, significantly reduced itch severity in five out of eight patients. We have previously reported that nalfurafine inhibits scratching in rats with cholestasis induced by chronic ethylene estradiol injections [42]. In a recently developed mice model of cholestasis (partial ligation of bile duct), authors reported that naloxone, U50,488, and clonidine (an α2-adrenoceptor agonist) significantly reduced scratching [43].

CQ has been shown to cause scratching in both humans and in rodents. CQ induce itch in healthy volunteers [44] and in patients during the treatment of malaria [45]. CQ elicits scratching behavior in rats [46] and in mice [29,47]. It was shown that CQ binds to Mas-related G-protein coupled receptor (Mrgprs) A3/X1 [48]. Multiple mediators and receptors are also involved in CQ-induced itch and are accepted as non-histaminergic itch [30]. Previously, we reported that CQ elicited-scratching was significantly reduced by pretreatment with nalfurafine in mice [29]. Munanairi et al. [49] reported that KOR and GRPR overlap at the spinal cord and activation of KOR inhibits GRPR-mediated itch in mice. Here, we showed that nalbuphine also inhibits CQ-induced scratching and antiscratch activity of nalbuphine is through KORs in mice.

In conclusion, nalbuphine is effective to reduce the itch like behavior induced by the TAT-HIV-1 protein (a protein is responsible for transcription of viruses and infection), DCA (one of the mediators responsible for cholestatic pruritus), and CQ in mice model acute itch. Since all these three conditions cause clinical itch in humans, we suggest that nalbuphine, a drug that has been in clinical use for over 40 years and is not a scheduled agent, will be effective for treating chronic itch in humans. Nalbuphine is already in clinical studies for cholestasis, but a clinical trial for chronic itch in HIV patients could be added.

## 4. Materials and Methods

### 4.1. Animals

Male Swiss–Webster mice (Taconic Biosciences, Germantown, NY, USA) and male C57BL/6J WT and KOR KO mice (007558-B6.129S2-Oprk1tm1Kff/J, Jackson Laboratories, Bar Harbor, ME, USA) weighing 25–30 g were used. WT and KOR KO mice were generated by homozygous breeding. Animals were housed in a temperature- and humidity-controlled environment with a 12-hr light–dark cycle. They were supplied with food and water ad libitum. Before any procedure was initiated, the mice were acclimated for a week in the animal facility. Behavioral testing was performed between 11:00 A.M. and 5:00 P.M. All animal care and experimental procedures were approved by the Institutional Animal Care and Use Committee of Temple University (protocol number 5021), conducted according to the NIH Guide for the Care and Use of Laboratory Animals. Between 6 and 8 animals/group were used for experiments.

### 4.2. Observation of Scratching Behavior

Acute scratching mouse model of itch described previously by Kuraishi et al. [50] was used. Mice were acclimated individually in rectangular observation boxes for at least an h before any injection or observation. Following acclimation, mice were injected s.c. in the area behind the neck with either saline or TAT-HIV-1 (0.1–1 mg/kg), deoxycholic acid (DCA, 0.3–10 mg/kg) to examine and develop dose–responses. One min after the injections, mice were observed for 30 min and the number of hindleg scratches directed to the back of the neck was counted by an observer. Since we have previously reported dose–response for CQ, we only used a submaximal dose (10 mg/kg) of CQ for the studies. To study whether nalbuphine would inhibit scratching induced by TAT-HIV, DCA, or CQ, mice were pretreated with nalbuphine (0.3–10 mg/kg, s.c.) at −30 min. Then, they were administered a fixed dose (submaximal dose) of pruritogen behind the neck area and they were observed, and scratching bouts were counted.

To study whether nalbuphine alleviates scratching acting on KORs, both genetic and pharmacologic approach was used to eliminate KORs. For the genetic approach, KOR knock out (KO) and wildtype (WT) littermates of C57BL/6J mice were used. CQ was chosen as pruritogen for this aim. Nalbuphine at 10 mg/kg, which significantly reduces scratching in Swiss–Webster, mice did not inhibit scratching induced by CQ in C57BL/6J mice. Then, we tried nalbuphine at 20 mg/kg, and we did not observe any behavioral depression following administration. Mice were injected with nalbuphine or saline and 30 min later they were administered CQ behind the neck and the number of scratches was counted for 30 min. For pharmacologic approach, Swiss–Webster mice were pretreated with either saline or norbinaltorphimine (nor-BNI, 20 mg/kg, intraperitoneally) at −20 h. The next day, mice were administered either saline or a fixed dose of nalbuphine (10 mg/kg). Thirty min later, saline or CQ was injected behind the neck of the mice. One min following injection, the scratching bouts were counted for 30 min.

### 4.3. Chemicals

Nalbuphine HCl, deoxycholic acid, and chloroquine were purchased from Sigma-Aldrich (St. Louis, MO, USA) and dissolved in saline. TAT-HIV-1 (32–62) was a generous gift from Phoenix Pharmaceuticals (Burlingame, CA, USA) and dissolved in saline. Compounds were administered as 0.1 mL/10 g body weight.

## Data Availability

Not applicable.

## Sample Availability

Samples of the compounds are not available from the authors.
